# Topography and Nonlinear Optical Properties of Thin Films Containing Iodide-Based Hybrid Perovskites

**DOI:** 10.3390/nano14010050

**Published:** 2023-12-23

**Authors:** Agnieszka Marjanowska, Houda El Karout, Dominique Guichaoua, Bouchta Sahraoui, Przemysław Płóciennik, Anna Zawadzka

**Affiliations:** 1 Institute of Physics, Faculty of Physics, Astronomy and Informatics, Nicolaus Copernicus University in Torun, Grudziadzka 5, 87-100 Torun, Poland; 2 Centre for Modern Interdisciplinary Technologies, Nicolaus Copernicus University, Wilenska 4, 87-100 Torun, Poland; przemas@fizyka.umk.pl; 3 LPhiA, SFR Matrix, University of Angers, Bd Lavoisier 2, 49045 Angers CEDEX 2, France; houda.elkarout@univ-angers.fr (H.E.K.); dominique.guichaoua@univ-angers.fr (D.G.); bouchta.sahraoui@univ-angers.fr (B.S.); 4 MOLTECH-Anjou-UMR CNRS 6200, SFR MATRIX, University of Angers, 49000 Angers, France; 5 Institute of Engineering and Technology, Faculty of Physics, Astronomy and Informatics, Nicolaus Copernicus University in Torun, Grudziadzka 5, 87-100 Torun, Poland

**Keywords:** CH_3_NH_3_*M*I_3_ hybrid perovskite, thin films, transmission, atomic force microscopy, nonlinear optical effects

## Abstract

This article covers selected properties of organic–inorganic thin films of hybrid perovskites with the summary formulas CH_3_NH_3_*M*I_3_, where *M* = Pb, Cd, Ge, Sn, Zn. The paper discusses not only the history, general structure, applications of perovskites and the basics of the theory of nonlinear optics, but also the results of experimental research on their structural, spectroscopic, and nonlinear optical properties. The samples used in all presented studies were prepared in the physical vapor deposition process by using co-deposition from two independent thermal sources containing the organic and inorganic parts of individual perovskites. Ultimately, thin layers with a thickness of the order of nanometers were obtained on glass and crystalline substrates. Their structural properties were characterized by atomic force microscopy imaging. Spectroscopic tests were used to confirm the tested films’ transmission quality and determine previously unknown physical parameters, such as the absorption coefficient and refractive index. Experimental results of the nonlinear optical properties were obtained by studying the second and third harmonic generation processes and using initial sample polarization in the so-called Corona poling process. The obtained experimental results allowed us to determine the second- and third-order nonlinear optical susceptibility of the tested materials.

## 1. Introduction

Perovskites have gained immense popularity in photovoltaics over the last 15 years. Their exceptional ability to absorb electromagnetic radiation within the 300–800 nm range has led to their widespread adoption in the production of thin-film photovoltaic cells. This contributed to a significant increase in photovoltaic efficiency. Moreover, ongoing research into perovskites has revealed their intriguing nonlinear effects, opening up new avenues of exploration. These nonlinear properties have the potential for diverse applications, including laser frequency conversion. The combination of unique optoelectronic and nonlinear properties in the perovskite materials have made it a promising material that can significantly improve the performance of optoelectronic devices. The discovery of such properties has sparked considerable interest among researchers and scientists, leading to innovative developments in the realm of optics and photonics [1,2]. 

**Figure 1:**
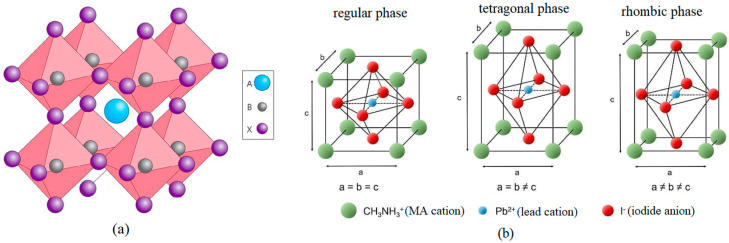
(**a**) The ideal crystal structure of a perovskite, (**b**) changes in the perovskite crystal structure depending on their temperature.

The name perovskite comes from the name of the Russian mineralogist Lev Perovsky. The first scaled perovskite was calcium titanate CaTiO_3_. It was discovered in 1838 in the Ural Mountains by Gustav Rose. Materials with the general structural formula ABX_3_ are called perovskites. In place A, there can be almost 30 elements: metal cations from the first or second group of the periodic table or transition metal cations. Element B can be about 50 elements, which are cations with a coordination number of 6, and X can be replaced by an oxide, sulfide, or halide anion [3]. When a halide anion is present in the X position (X = Cl, Br, I), the perovskite is called a halide perovskite. The ideal structure of the perovskite crystal lattice comprises regular octahedrons connected to each other, in which six X anions surround the B cation in such a way that they form an octahedron. In turn, there is a cation A between the single octahedra, whose task is to balance the charge of the crystal lattice (Figure 1a). 

The stability of the perovskite crystal structure depends on its temperature. It was tested that, depending on the temperature, these materials can exist in three phases: tetragonal—at room temperature, rhombic and regular—at temperatures lower and higher than room temperature, respectively [4]. Figure 1b shows the regular phase of a single perovskite atomic cell. The distances between adjacent methylammonium atoms are marked as *a*, *b*, and *c*. In the case of the regular phase, the relationship *a* = *b* = *c* is met between the distances *a*, *b*, and *c*, for the tetragonal phase *a* = *b* ≠ *c*, and in the case of the rhombic phase *a* ≠ *b* ≠ *c*. The stability of the perovskite’s crystal structure and its crystal lattice’s distortion can be determined by calculating the value of the tolerance factor *t* (Equation (1)). For a perovskite structure with a stable regular phase, the condition 0.9 < *t* < 1 is met; in the case of a distorted crystal structure 0.8 < *t* < 0.9, while the value of t greater than 1 or less than 0.8 usually indicates the presence of non-perovskite [5].

(1) $$t=\frac{r_{A}+r_{X}}{\sqrt{2}r_{B}+r_{X}}$$

where $r_{A}$, $r_{B}$, $r_{X}$ denotes the radii of the corresponding ions. 

Perovskite is a material that occurs in the natural environment, but it can also be obtained through chemical synthesis. The possibility of chemical synthesis of the perovskite is one of its advantages because, thanks to it, it is possible to obtain a material with a selected composition, i.e., it is possible to manipulate the selection of elements *A*, *B*, *X* to a certain extent, and thus influence its structural, dielectric, ferroelectric, piezoelectric, and optical properties, e.g., modulation of the threshold wavelength of the absorbed light or the stability of the thin layer. The optoelectronic properties distinguish perovskite from other materials, making them promising for optoelectronic applications. The properties result from a particular and unique structure of the crystal lattice. The properties of each perovskite are determined by the elements *A*, *B*, *X*. The characteristic features of the group of perovskite materials are the outstanding ability to absorb electromagnetic radiation in the wavelength range of 300–800 nm, high absorption coefficient α, long lifetime of carriers, high mobility of holes and electrons compared to other organic materials [6,7], mixed type of conductivity and instability at high humidity and high temperature. The instability of perovskites is their main disadvantage that has stopped the commercialization of devices containing perovskites [8]. 

Interest in hybrid perovskites, i.e., organic–inorganic, had grown enormously over the last 15 years when in 2009, their potential application in photovoltaics was presented [9]. Within a short time, it was possible to obtain a perovskite photocell with efficiency of 22% [10,11]. For several years, material sciences and engineering have been looking for a way to produce time-stable and efficient photovoltaic cells that will stand out from the most popular silicon cells in terms of achieved efficiency and production costs. However, it should be remembered that perovskites are materials that can be used not only in photovoltaics, but also in nonlinear optics and their applications.

Materials exhibiting nonlinear optical effects (NLO) have become invaluable in numerous practical applications, spanning from industrial manufacturing to advanced microsurgery techniques. Thanks to the progress in nonlinear optics and materials engineering as well as the interdisciplinary work of research groups, it is possible to have a compact pocket laser pointer or to transmit data quickly and reliably. The pursuit of nonlinear optical effects in novel materials holds profound significance for scientific advancement. Through years of dedicated research, materials demonstrating these effects have found diverse applications among others, in optical computing [12,13], optical data storage [14], optical fiber communication [15], optical switches [16], light generation and modulation [17], optical parametric amplification [18], laser frequency conversion [19], ultrafast pulse measurement [20], modulators [21], material analysis [22], photodynamic therapy [23], dynamic image processing [24] or high-resolution imaging [25]. 

In this paper, linear and nonlinear optical properties and structural properties of thin films of selected perovskites CH_3_NH_3_*M*I_3_, where *M* = Pb, Cd, Ge, Sn, Zn were described. The materials differ only in the *M* member, which is a metal cation. The common parts are the methylammonium iodide and iodide, which is a halogen anion. It is primarily the change in the *M* atom in the above perovskite structures that affects their optoelectric properties. An innovative production technology based on the physical vapor co-deposition (PVco-D) process was used to obtain thin layers of investigated hybrid perovskites. This modern technology enabled the precise fabrication of thin layers with specific thickness, ensuring uniformity across a large surface area. This uniformity is of paramount importance in practical applications, enhancing the reliability and performance of devices utilizing these materials.

## 2. Materials and Methods

### 2.1. Materials

The perovskites studied in this work are hybrid organic–inorganic halogen perovskites such as CH_3_NH_3_PbI_3_, CH_3_NH_3_CdI_3_, CH_3_NH_3_GeI_3_, CH_3_NH_3_SnI_3_, CH_3_NH_3_ZnI_3_. Thin layers of the above materials were created by combining two solid substrates, i.e., the methylammonium iodide part (CH_3_NH_3_I) and the metal iodide part (PbI_2_, CdI_2_, GeI_2_, SnI_2_, ZnI_2_) in the process of physical vapor deposition. The methylammonium iodide part was obtained by chemical synthesis [26], and the high purity metal iodides used to create the perovskites were purchased from Sigma-Aldrich (Poznań, Poland). Thermo Scientific MENZEL-GLASER (Karlsruhe, Germany) glass plates with a surface area of about 2 cm^2^ were used as substrates for thin layers of perovskites.

### 2.2. Deposition Technique of Thin Films

**Figure 2:**
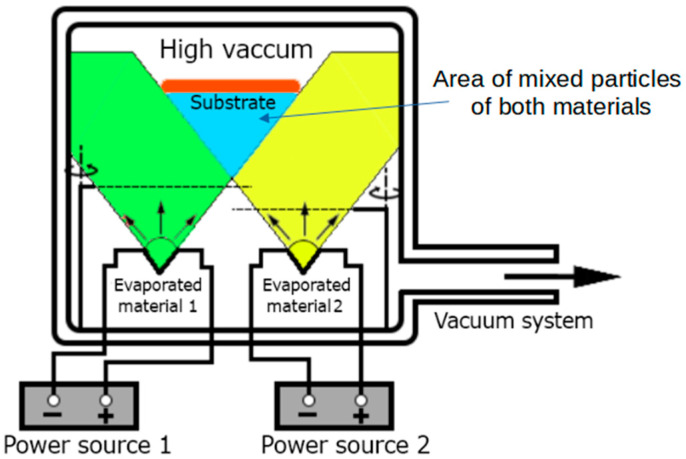
The idea of the co-deposition process.

The investigated perovskites were formed using the physical vapor deposition (PVD) process using the NANO 36^TM^ vacuum sputtering machine (Kurt J. Lesker Company, Dresden, Germany) [27]. The PVD process of the described materials took place in a sealed vacuum chamber, under controlled conditions of temperature and pressure, at a pressure of about 10^−5^ Torr. The Thin Film Deposition System is equipped with two independent power sources, enabling the seamless execution of the co-deposition process. The process of co-deposition comprises the simultaneous evaporation from the sources and the deposition of two or more chemical compounds on the substrate. The molecules of the evaporated compounds mix inside the vacuum chamber and settle on the substrate, creating a structure with physicochemical properties different from the properties of the component materials. In this way, thin layers of doped semiconductors and perovskites can be obtained, which in this work are obtained by mixing pairs of the methylammonium iodide part with the appropriate metal iodide. The concept of the co-deposition process and the interior of the vacuum chamber are shown in Figure 2. Inside the vacuum chamber, in its lower part, there are two independently powered vapor sources, and in its upper part, a rotating plate on which clean substrates are placed. The distance between the steam sources and the plate is 20 cm. Between them, there are two shutters and two piezoelectric sensors, allowing for precise control over the layer thickness and application speed. The steam sources are ceramic baskets with a nozzle of 10 mm in diameter on the top. The substrates are placed in them separately. After obtaining the appropriate pressure conditions, the vapor sources are independently heated until sublimation of both materials begins, then the shutters are opened and the co-deposition process begins. The plate with the substrates is rotated at a speed of 15 rot/min throughout the co-deposition. After obtaining the appropriate layer thickness, the co-deposition process ends and the shutters are closed. To obtain a thin layer by the above-described system, the deposition process must be programmed with the necessary deposition parameters. The values of these parameters for individual chemical compounds are listed in Table 1. 

Utilizing the co-deposition technique for thin film production proves immensely advantageous. This method is particularly beneficial when dealing with diverse materials housed in separate sources, necessitating specific temperatures for their sublimation. The beauty of this approach lies in the utilization of distinct power and steam sources, enabling precise temperature control for each material. Furthermore, the PVD technique plays a pivotal role in ensuring the quality of thin layers. It facilitates the creation of high-quality films that uniformly coat the substrate across its entire surface. Additionally, the application process under high vacuum conditions enhances the purity of the materials, contributing significantly to the overall quality and integrity of the thin films produced [28].

### 2.3. Surface Characterization of Thin Films

Nanosurf EasyScan 2 atomic force microscope system was used to study the surface topography of investigated perovskite thin films. The microscope was working in contact mode with the Sicon-A cantilever. The resulting 3D images were analyzed using Gwyddion 2.58 software [29]. The layer profiles were checked, the average roughness was calculated, and Minkowski functionals were determined, on the basis of which it is possible to analyze the spatial location of holes (or valleys) on the surface of the tested sample. 

Of the Minkowski functionals, three stand out: Minkowski Volume, Minkowski Boundary, and Minkowski Connectivity [30]. In order to determine the functionals, the h parameter is introduced, which is the height relative to the ground at which the topography of the layer is analyzed. The next parameters are *N_B_* and *N_W_*, which mean material points and empty points (air), respectively, *N_Bounded_*—limited pixels, $n_{B}$ and $n_{W}$—the number of isolated areas or islands at height *h*. The first of the functionals—Minkowski Volume determines the amount of matter at the level *h* and is defined as follows:

(2) $$V\left(h\right)=\frac{N_{B}}{N_{B}+N_{W}}.$$

Minkowski Boundary (Equation (3)) and Minkowski Connectivity (Equation (4)) describe the contour length and surface microstructure at the *h* level, as follows:

(3) $$B\left(h\right)=\frac{N_{Bounded}}{N_{B}+N_{W}}$$

(4) $$\chi \left(h\right)=\frac{n_{B}-n_{W}}{N_{B}+N_{W}}$$

### 2.4. Spectroscopic Measurements

Spectroscopic measurements were carried out in the wavelength range of 270–1050 nm using the Shimadzu UV-1800 spectrometer (Kyoto, Japan). They were used in estimates of the refractive index and the linear absorption coefficient associated with the calculation of nonlinear optical effects. 

### 2.5. NLO Measurements

Perovskite thin films were examined to explore their optical nonlinear effects such as second harmonic generation (SHG) and third harmonic generation (THG). To enhance the quality of the SHG signal, Corona poling was implemented. These measurements of nonlinear optical effects were conducted using the Maker fringe technique, and the gathered data were analyzed using the theoretical comparative models.

#### 2.5.1. Interaction of Matter with a Strong Electromagnetic Field—The Process of Generating Higher Harmonics

Strong electric fields interacting with matter change their properties nonlinearly, which means that the electric polarization of the medium in which such an electric field propagates will depend disproportionately on it. It follows that the electric polarization *P* of a medium affected by a strong electric field E will be the sum of the linear and nonlinear polarizations (Equation (5)):

(5) $$P\left(r,t\right)=P_{N}\left(r,t\right)+P_{NL}\left(r,t\right)=\chi_{e}^{1}E\left(r,t\right)+\chi_{e}^{2}E{\left(r,t\right)}^{2}+\chi_{e}^{3}E{\left(r,t\right)}^{3}+...+\chi_{e}^{N}E{\left(r,t\right)}^{N}$$

where:

*χ_e_*^(1)^—linear electrical susceptibility of the first order, describing the linear optical phenomena, 

*χ_e_*^(*N*)^—N-order nonlinear electrical susceptibility, describing the N-order nonlinear effects. 

The situation is similar to the magnetic polarization M of the medium, which in the case of the interaction of a strong magnetic field H with matter, is the sum of linear and nonlinear polarization. In the case of laser light, which is an electromagnetic wave of enormous intensity interacting with a material medium, we observe the appearance of electric and magnetic polarization, both linear and nonlinear. The effect of this is the observation of optical linear and nonlinear effects in the material medium. The first group includes optical phenomena such as reflection, refraction, and radiation scattering. The second group comprises the Pockels effect, optical Kerr effect, generation of the second and higher harmonics. Nonlinear optical phenomena were first observed in the 1960s, shortly after Maiman discovered the first ruby laser [31,32]. After that, interest in nonlinear optics increased enormously and continues to this day [33,34,35]. 

#### 2.5.2. Theoretical Models for Higher Harmonic Generation Analysis

The values of nonlinear electrical susceptibilities are calculated on the basis of experimentally obtained higher harmonic spectra and by using theoretical models in the calculations. The choice of the theoretical model used during data analysis depends on, e.g., the type of samples tested and their physical state. Of several theoretical models, two were used in this work—the Lee model and the Kubodera–Kobayashi model [36,37,38]. 

To determine the value of the second-order nonlinear electrical susceptibility, Lee’s theoretical model was used. In this model, using spectra determined experimentally for the tested material and the reference material, the maximum amplitude of the intensity of the second harmonic of the tested nonlinear material is directly compared with the maximum amplitude of the reference material. Thanks to this procedure, it is possible to estimate the order of effective second-order electrical susceptibility *χ*^2^, which is expressed by the formula:

(6) $$\chi^{2}=\chi_{ref}^{2}\frac{2}{\pi }\frac{L_{Cref}}{d}\frac{\frac{1}{2}\alpha d}{1-e^{\frac{-1}{2}\alpha d}}\sqrt{\frac{I_{2\omega }}{I_{2\omega ref}}}$$

where:

$\chi_{ref}^{2}$—second-order nonlinear susceptibility of the reference material;

$L_{Cref}$—coherence length of the reference material;

d—thickness of the thin layer; 

α—linear absorption coefficient of the material for the fundamental wavelength =1064 nm; 

$I_{2\omega }$—intensity of the second harmonic of the tested material; 

$I_{2\omega ref}$_—_intensity of the second harmonic of the reference material.

The reference material for the measurement of nonlinear electrical susceptibility was a quartz glass with a thickness of 1 mm for which the $\chi_{ref}^{2}$ = 1 pmV^−1^. 

Aiming to estimate the order of the value of the third-order nonlinear electrical susceptibility $\chi^{3}$, the Kubodera–Kobayashi model was used. As in the case of second-order nonlinear electrical susceptibility calculations, in this case the maximum amplitude of the third harmonic intensity of the tested nonlinear material is directly compared with the maximum amplitude of the reference material (Equation (7)). This time the reference material is silica glass with a thickness of 1 mm for which $\chi_{ref}^{3}$= 2 × 10^−22^ m^2^ V^−2^ was adopted.

(7) $$\chi^{3}=\chi_{ref}^{3}\frac{2}{\pi }\frac{L_{Cref}}{d}\frac{\frac{1}{2}\alpha d}{1-e^{\frac{-1}{2}\alpha d}}\sqrt{\frac{I_{3\omega }}{I_{3\omega ref}}}$$

where:

$\chi_{ref}^{3}$—third-order nonlinear susceptibility of the reference material;

$L_{Cref}$—coherence length of the reference material; 

d—thickness of the thin layer; 

α—linear absorption coefficient of the material for the fundamental wavelength =1064 nm; 

$I_{3\omega }$—intensity of the third harmonic of the tested material; 

$I_{3\omega ref}$—intensity of the third harmonic of the reference material. 

#### 2.5.3. SHG and THG Apparatus 

**Figure 3:**
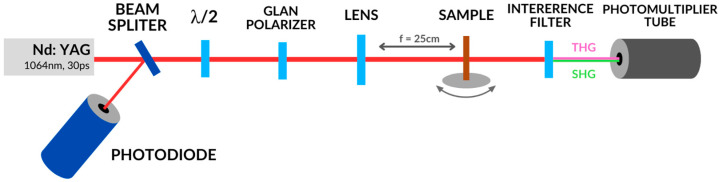
SHG and THG measuring system.

The diagram of the apparatus for carrying out SHG and THG measurements is shown in Figure 3. In the presented work, the measurement of SHG and THG signals is carried out using an apparatus that differs only in the interference filter, which is placed directly in front of the photodetector. The presented measurement method is one of several known methods for measuring third-order nonlinear optical effects. Nonlinear third-order effects can also be investigated using a technique called impulsive stimulated raman spectroscopy (ISRS) [39,40,41].

The source of laser radiation in the system used is a pulsed Nd:YAG laser (Ekspla, PL2250) with a pulse duration of 30 ps. It is a laser with a wavelength of 1064 nm and a frequency of 10 Hz (Table 2). The radiation beam coming from the laser hits the beam splitter. Part of the radiation is reflected in this place and falls into the photodiode. Part of the laser beam passes through the splitter and reaches the half-wave plate (λ/2) and the Glan polarizer. Thanks to these two elements, it is possible to change the polarization of the radiation and thus change its energy. The appropriately polarized beam (S or P) reaches a focusing lens with a focal length of 25 cm and then falls on the test sample. The beam that passes through the sample reaches the interference filter. The filter is used to cut out unwanted wavelengths from the primary beam. Thanks to this, only the valuable signal corresponding to the generation of higher harmonics reaches the photomultiplier. In the case of SHG, these are wavelengths of 532 nm, and for THG—355 nm. The sample in the form of a thin layer applied to a glass substrate with a thickness of 1 mm is located between the focusing lens and the interference filter. It is placed on a rotating table in a place where the axis of rotation of the table is very close to the focal point of the lens. This precise alignment gives the laser beam incident on the sample the highest energy. During the measurement, the sample rotates relative to the direction of the incident beam from −60° to +60° with 0.5° steps. The signal is collected and averaged for each measurement point. The output is the result, which is a dependence of the signal intensity of the generated higher harmonic in the incident angle function. 

**Figure 4:**
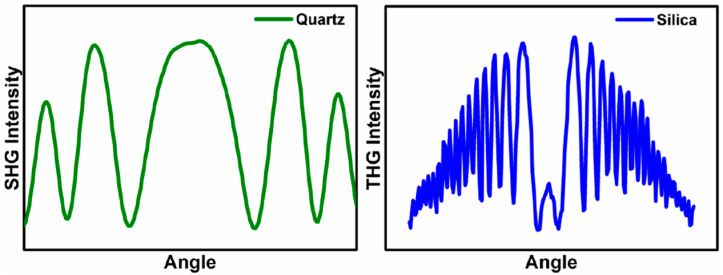
Typical harmonic measurement results for reference materials.

Each measurement series begins with the measurement of reference materials for SHG and THG, which are Y-cut quartz crystal and silica glass, respectively, in the form of 1 mm thick plates. Typical measurement results for reference materials are schematically shown in Figure 4. For this type of measurement, it is important to maintain the symmetry of the signal with respect to the 0° angle. The results obtained for the tested samples are compared with the corresponding results for reference materials. On this basis and thanks to the use of Lee and Kubodera–Kobayashi theoretical models, the nonlinear electrical susceptibility of the tested materials is calculated.

#### 2.5.4. Corona Poling Technique

**Figure 5:**
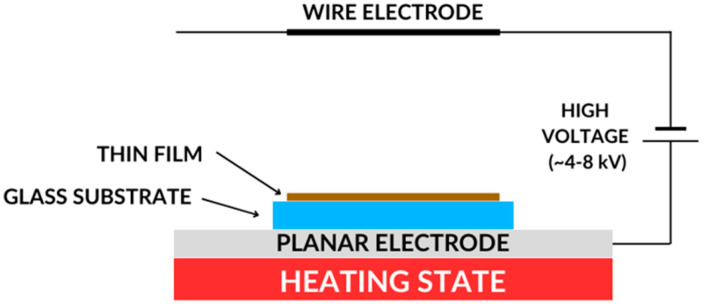
Corona poling technique scheme.

The generation of the second harmonic is closely related to the symmetry of the tested material—the SHG signal is observed only for non-centrosymmetric materials. In the case of centrosymmetric materials, it is possible to produce macroscopic non-centrosymmetry in the test sample. This is carried out using the Corona poling method (Figure 5). Thanks to this method, it becomes possible to observe the SHG signal for a sample for which this signal has not been recorded before. 

The Corona poling method relies on forcing a change in the orientation of the molecules that make up the sample and have dipole moments by applying an external, strong electric field. In the first stage of the process of changing the orientation of molecules, the sample is heated to a temperature of about 95 °C, and then an external and strong electric voltage of about 6.5 kV is applied. Thanks to this, the molecules are arranged along the applied electric field lines and ordered. When the external electrical voltage is turned off, the molecules remain in order. The time of this arrangement depends on the individual chemical properties of the tested material. 

## 3. Results and Discussion

The tested materials are thin layers of halide perovskites CH_3_NH_3_PbI_3_, CH_3_NH_3_CdI_3_, CH_3_NH_3_GeI_3_, CH_3_NH_3_SnI_3_, CH_3_NH_3_ZnI_3_ deposited on glass substrates. The thicknesses of individual layers are listed in Table 4. All samples were obtained by PVD. 

### 3.1. Transmittance

**Figure 6:**
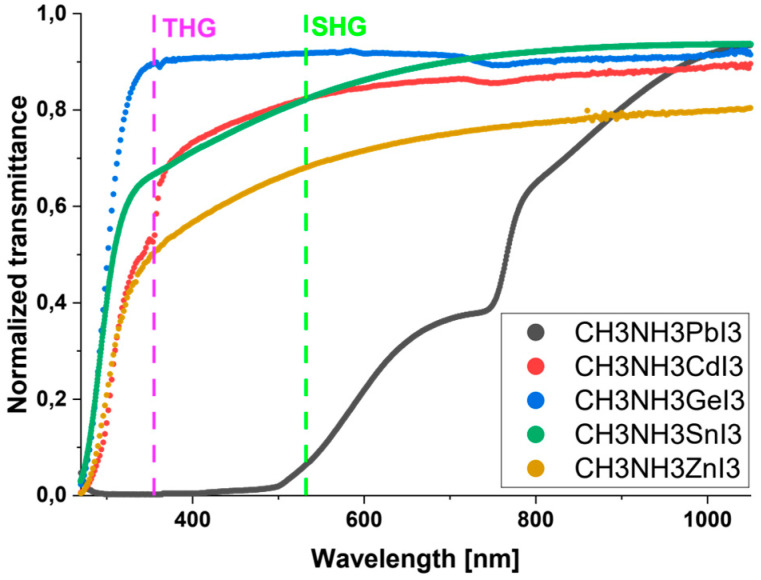
Normalized UV-Vis-NIR transmittance spectra of investigated perovskites with THG (355 nm) and SHG (532 nm) wavelengths.

Optical and normalized transmittance spectra of the tested samples of perovskite thin films are presented in Figure 6. The thicknesses of individual layers are in Table 4. Measurements were made immediately after the attainment of thin layers. Based on the above, the least transmitting material is the CH_3_NH_3_PbI_3_ perovskite. The perovskite CH_3_NH_3_GeI_3_ is almost transparent in the investigated wavelength range. The transmittance spectrum of the first of these materials is characterized by maxima at wavelengths of 717 nm, 794 nm, and 1020 nm. It shows a broad absorption band in the range from about 300 nm to almost 1000 nm, thanks to which it can be successfully used in the field of photovoltaic applications. The first maxima (717 nm) directly characterizes the CH_3_NH_3_PbI_3_ perovskite, while the next two maxima (794 nm and 1020 nm) result from the phenomenon of interference of radiation reflected at the layer-substrate boundary. The location of these two maxima is closely related to the thickness of the tested layer [42].

The transmittance spectra of the remaining perovskites have a flattened shape, and their transmittance in the examined wavelength range varies from about 50% to about 90%. With respect to NLO, in Figure 6, there are also SHG and THG lines corresponding to the wavelengths of 532 nm and 355 nm, respectively. Knowledge of the absorption of individual materials at these wavelengths is necessary to calculate the absorption coefficients and, later, the nonlinear optical properties of SHG and THG. For nonlinear studies, the tested samples must not absorb the fundamental wavelength of the laser 1064 nm. The values of the absorption coefficients calculated based on the transmittance spectrum are presented in Table 3.

### 3.2. AFM Analysis

**Figure 7:**
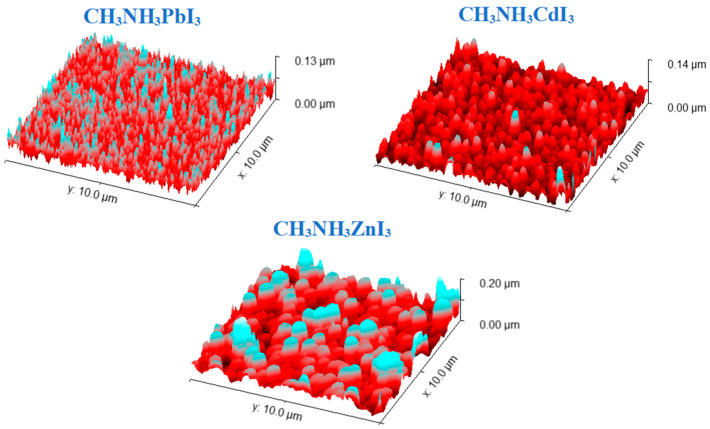
AFM 3D images of perovskite thin films: CH_3_NH_3_PbI_3_, CH_3_NH_3_CdI_3_, CH_3_NH_3_ZnI_3_.

**Figure 8:**
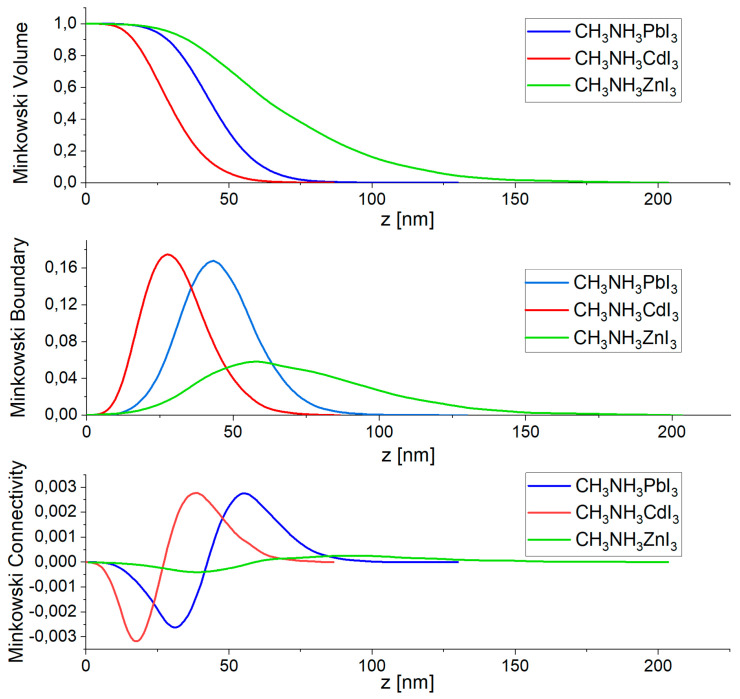
Minkowski functionals of the halide perovskite thin film: Minkowski Volume, Minkowski Boundary, Minkowski Connectivity.

The detailed surface topography of selected perovskite thin films was studied by contact mode AFM. The tested samples were deposited on glass substrates. The obtained images were analyzed using the Gwyddion software and the Minkowski Functional method. The 3D images of the analyzed thin-film structures are shown in Figure 7, and the graphical results of the Minkowski Functional analysis are shown in Figure 8. Table 4 shows the average height of the crystallites forming the layer and its average roughness calculated based on surface topography images. The analyzed thin-film surfaces are 10 µm × 10 µm. All the tested thin layers are characterized by almost uniform coverage of the entire tested surface. Single deviations in the coverage regularity can be seen in the form of single slightly higher (in the case of CH_3_NH_3_CdI_3_) or lower crystallites (in the case of CH_3_NH_3_ZnI_3_). The most uniform structure has a thin layer of perovskite CH_3_NH_3_CdI_3_, which is confirmed by the lowest value of the average roughness. The most important from the point of view of application and subsequent measurements is the fact that the entire tested surface is covered with a thin layer of material. The nature of the obtained thin-film structures is reflected in the results of the Minkowski Functional analysis. The MV characteristics have a smooth shape, which proves that the formation of thin layers in the process of co-deposition proceeds harmoniously over the entire surface. The formed crystallites have the form of sharper needles for the CH_3_NH_3_PbI_3_ perovskite and the form of mild structures in the case of CH_3_NH_3_ZnI_3_. The MC characteristic, in turn, reveals the nature of the created layer—the negative part of the graph refers to its porosity, and the positive part to its islandness. Moreover, the maximum of this characteristic is equivalent to the maximum density of islands and the minimum to the maximum density of valleys in the layer. 

**Table 4:** Thicknesses of perovskite thin layers read from the vacuum sputtering machine and the average height of crystallites and average roughness of the layers calculated on the basis of surface topography measurements using AFM.

Material	Thickness [nm]	Average Crystallite Height [nm]	Medium Roughness [nm]
CH_3_NH_3_PbI_3_	300.0	45.2	13.2
CH_3_NH_3_CdI_3_	175.0	32.7	12.5
CH_3_NH_3_GeI_3_	70.0	−	−
CH_3_NH_3_SnI_3_	253.0	−	−
CH_3_NH_3_ZnI_3_	260.0	71.0	30.8

### 3.3. SHG and THG Results

**Figure 9:**
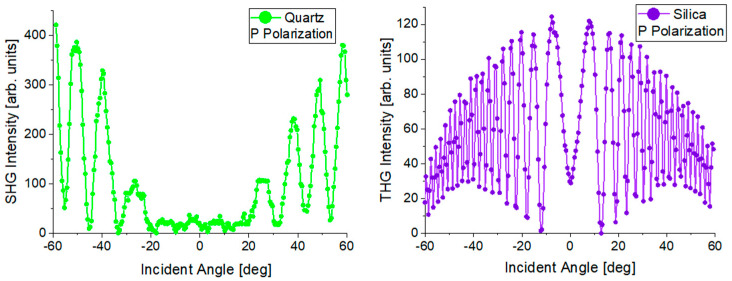
SHG and THG measurement results of reference materials.

**Figure 10:**
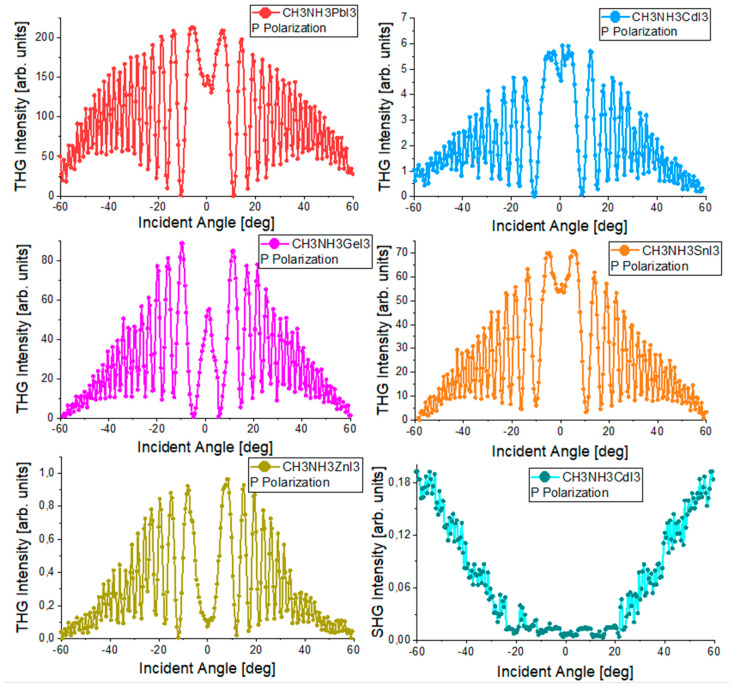
Graphical results of THG and SHG (after Corona poling) measurements of the tested perovskites.

Measurements of the nonlinear optical effects of SHG and THG were made using the apparatus shown schematically in Figure 3. Quartz glass for SHG measurements and silicon glass for THG were used as reference materials. The measurement results of reference materials are shown in Figure 9. SHG measurements were carried out for laser radiation with P and S polarization, and THG measurements were carried out for P polarization. To improve the SHG signal, the Corona poling technique was used. The collected THG and SHG signals of the tested materials are presented graphically in Figure 10. The results of SHG and THG calculations, as well as values for the relevant reference materials, are presented in Table 5. The analysis of the obtained data was based on the theoretical Kubodera–Kobayashi model (for THG) and the Lee model (for SHG). 

The spectra of both reference materials are symmetrical at about the 0° point, which means that the measurement system was correctly calibrated in the first stage of the measurements. The THG measurement results of the tested perovskites presented in Figure 10 also show symmetry. The observed symmetry proves the good quality and uniform thickness of the thin layers produced by the PVD technique. The THG generation process does not depend on the polarization of the incident beam. Therefore, Figure 10 contains the results only for the P polarization. The intensity of the observed signal depends on the angle of incidence of the laser radiation on the sample. None of the examined perovskite thin films revealed the generation of a second-order nonlinear signal. Therefore, the Corona poling method was used. After this treatment, only one of the perovskites showed a signal confirming the occurrence of second-order nonlinear effects. This signal is presented in Figure 10. By comparing the obtained spectra of higher harmonics with the spectra of reference materials, the values of SHG and THG were calculated. The strongest THG signal was recorded for the CH_3_NH_3_PbI_3_ perovskite, and the calculated nonlinear electrical susceptibility χ^(3)^ is approximately (118 × 10^−22^)m^2^ V^−2^. CH_3_NH_3_CdI_3_ and CH_3_NH_3_GeI_3_ perovskites have very similar χ^(3)^ values. The weakest THG properties among the tested materials were observed for CH_3_NH_3_ZnI_3_. To compare the THG results with the results from other scientific works that focused on examining the THG effects of perovskite materials with similar structures, we can refer to perovskites (C_4_H_9_NH_3_)_2_PbI_4_ whose χ^(3)^ = 3.5 × 10^−18^ m^2^ V^2^ [43], CH_3_NH_2_/PbBr_2_: 1/1 whose χ^(3)^ = 1.25 × 10^−13^ m^2^ V^2^ [44]. The difference between the obtained numerical values of χ^(3)^ for CH_3_NH_3_PbI_3_ and (C_4_H_9_NH_3_)_2_PbI_4_ perovskites is visible. The results differ by about two orders of magnitude. However, it should be noted that these are not identical thin-film perovskite structures. These are materials with similar crystal structures and compositions. The difference in the determined values of third-order nonlinear susceptibility is most likely also influenced by the technique of obtaining thin-layer structures and the measurement method itself. As for second-order nonlinear effects, they were observed only for CH_3_NH_3_CdI_3_ after Corona poling. However, the observed signal was small, and the calculated SHG value was only 0.06 pmV^−1^. The SHG signal was obtained only for one material, while other publications [45,46] prove that the generation of a second-order nonlinear response can also be observed in thin layers of CH_3_NH_3_GeI_3_ perovskite and other hybrid perovskites. The main reason for such differences in the obtained results is the method of obtaining the thin layer. The PVD method used in this work causes a centrosymmetric orientation of the molecules forming the layer, which makes it impossible to observe a second-order nonlinear signal. The Corona poling method also does not sufficiently change the symmetry of the tested films, which is why observing the SHG signal is difficult.

## 4. Conclusions

In conclusion, linear and nonlinear optical properties and surface topography of thin films of CH_3_NH_3_PbI_3_, CH_3_NH_3_CdI_3_, CH_3_NH_3_GeI_3_, CH_3_NH_3_SnI_3_, CH_3_NH_3_ZnI_3_ perovskites of various thicknesses were investigated. All thin layers were made using the PVD technique. UV-Vis-NIR spectroscopy shows that the perovskite absorbing the largest part of electromagnetic radiation is the perovskite with PbI_2_. The remaining perovskites absorb a small part of the radiation from the studied area. Atomic force microscopy (AFM) confirms that the obtained thin layers cover the entire examined surface. In addition, thanks to the MFs analysis, the nature of the created layers can be determined. Measurements of nonlinear optical effects have shown that the studied perovskites are characterized by strong third-order nonlinear effects. However, in most cases, they do not reveal second-order nonlinear effects. CH_3_NH_3_CdI_3_ was the only material tested that showed a useful SHG signal. 

## Figures and Tables

**Table 1:** PVco-D process parameters for the tested materials.

Material	Parameters			
	Density [gcm^−3^]	Z-Factor	Rate [As^−1^]	Melting Point [°C]
CH_3_NH_3_I	2.22	1	0.2	~145
PbI_2_	6.16			~400
CdI_2_	5.67			~390
GeI_2_	4.32			~460
SnI_2_	5.28			~320
ZnI_2_	4.74			~445

**Table 2:** Fundamental beam parameters.

Laser	Nd: YAG
Laser wavelength	1064 nm
Laser energy	95 µJ
Pulse duration	30 ps
Repetition rate	10 Hz

**Table 3:** Absorption coefficients of perovskite thin films calculated based on transmittance spectra for wavelength λ = 532 nm and λ = 355 nm.

Material	T_532 nm_ [%]	A_532nm_	T_355nm_ [%]	A_355nm_	α_532nm_ [cm^−1^]	α_355nm_ [cm^−1^]
CH_3_NH_3_PbI_3_	6.36	1.20	0.24	2.62	92,755.00	202,930.00
CH_3_NH_3_CdI_3_	82.34	0.08	53.05	0.28	11,215.00	36,587.00
CH_3_NH_3_GeI_3_	91.74	0.04	89.33	0.05	12,439.00	16,280.00
CH_3_NH_3_SnI_3_	82.25	0.08	66.63	0.18	7801.00	27,334.00
CH_3_NH_3_ZnI_3_	68.04	0.17	51.02	0.30	14,958.00	26,141.00

**Table 5:** Results of SHG and THG calculations.

Material	Lee Model		Kubodera–Kobayashi Model
	χ^(2)^ [pmV^−1^] after CP		χ^(3)^ [10^−22^ m^2^ V^−2^]
	P Polarization	S Polarization	
Quartz	1		-
Silica	-		2
CH_3_NH_3_PbI_3_	-	-	118.20 ± 3.97
CH_3_NH_3_CdI_3_	0.06 ± 0.01	-	71.49 ± 4.09
CH_3_NH_3_GeI_3_	-	-	71.12 ± 10.20
CH_3_NH_3_SnI_3_	-	-	47.73 ± 1.89
CH_3_NH_3_ZnI_3_	-	-	43.10 ± 2.08

## Data Availability

The data presented in this study are available upon request from the corresponding author. The data are not publicly available due to funding requirements.
